# ﻿Morphology, multilocus phylogeny, and toxin analysis reveal two new species of *Amanita* section *Amanita* (Amanitaceae) from China

**DOI:** 10.3897/mycokeys.118.141080

**Published:** 2025-06-09

**Authors:** Yu-Ting Su, Fei Xu, Ping Zhang, Peng-Tao Deng, Meng-Meng Lai, Zuo-Hong Chen

**Affiliations:** 1 School of life science and resources and environment, Yichun University, Yichun, Jiangxi 336000, China Hunan Normal University Changsha China; 2 College of Life Science, Hunan Normal University, Changsha, Hunan 410081, China Yichun University Yichun China; 3 Ningxia Center for Disease Control and Prevention, Yinchuan, Ningxia 750004, China Ningxia Center for Disease Control and Prevention Yinchuan China

**Keywords:** Ibotenic acid, morphology, muscimol, taxonomy

## Abstract

Globally, many species of Amanitasect.Amanita (Amanitaceae, Agaricales) cause poisoning after consumption. *Amanitaflavomelleiceps***sp. nov.** and *Amanitaparvisychnopyramis***sp. nov.**, two new species of A.sectionAmanita from China, are described here. Morphology and molecular phylogenetic analyses based on five genes (ITS, nrLSU, *RPB2*, *TEF1-α*, and *TUB2*) revealed these two taxa as distinct species. *Amanitaflavomelleiceps***sp. nov.** is characterized by a yellowish-to-yellow pileus covered by verrucose volval remnants; a subglobose basal bulb with shortly limbate; subglobose to broadly ellipsoid, inamyloid basidiospores. *Amanitaparvisychnopyramis***sp. nov.** is characterized by a brownish pileus covered by subconical volval remnants; an ovate basal bulb with a cream limbate; and subglobose-to-broadly ellipsoid, inamyloid basidiospores. Screening for the most notorious toxins by liquid chromatography tandem mass spectrometry (LC-MS/MS) revealed the presence of ibotenic acid (IBO) and muscimol (MUS) in *A.parvisychnopyramis* and the absence of these toxins in *A.flavomelleiceps*.

## ﻿Introduction

Amanitasect.Amanita Corner and Bas comprises about 150 described and accepted species globally (Cui et al. 2018; Su et al. 2022; Zhou et al. 2023) (http://www.amanitaceae.org/; accessed on 24 Sep 2024). The section is characterized by striate margins, persistent volval remnants on the pileus, the presence of a basal bulb, and inamyloid basidiospores (Yang 2005; Cui et al. 2018; Su et al. 2023). To date, 28 taxa (21 species, two varieties, and two forms) of A.sect.Amanita have been reported from China (Gilbert 1940, 1941; Jenkins 1978, 1986; Cui et al. 2018; Su et al. 2022; Zhou et al. 2023).

Some species of A.sect.Amanita are responsible for neuropsychiatric mushroom poisoning. In Europe and North America, *Amanitamuscaria* (L.) Lam., *A.pantherina* (DC.) Krombh., and other species of sect. Amanita have caused human and animal poisoning cases (Elonen et al. 1979; Tulloss and Lindgren 2005; Rossmeisl et al. 2006; Gonmori et al. 2011; Garcia et al. 2015; Brvar et al. 2016; Mikaszewska-Sokolewicz et al. 2016; Moss and Hendrickson 2018; Jobin et al. 2023). In China, several species of sect. Amanita, such as *A.concentrica* T. Oda, C. Tanaka & Tsuda, *A.melleiceps* Hongo, *A.rufoferruginea* Hongo, *A.parvipantherina* Zhu L. Yang, M. Weiss & Oberw., *A.subglobosa* Zhu L. Yang, *A.pseudosychnopyramis* Y.Y. Cui, Q. Cai & Zhu L. Yang and *A.sychnopyramis* Hongo have caused at least 110 poisoning cases in recent years (Li et al. 2019, 2020, 2021, 2022; Liu et al. 2020; Chen et al. 2022). The responsible toxins in these Amanitasect.Amanita species are isoxazole derivatives, primarily ibotenic acid (IBO) and muscimol (MUS). These toxins possess thermostability, and their consumption can cause glutamatergic neurotoxicity (Diaz 2005; Chen et al. 2014, 2016).

In this study, we describe two new species of A.sect.Amanita based on morphological and multigene phylogenetic evidence. We also identify the presence of IBO and MUS to facilitate prevention and clinical treatment of potential Amanitasect.Amanita poisoning accidents.

## ﻿Materials and methods

### ﻿Sample collection and morphological observation

Specimens were collected from Sichuan Province (28°19'15"N−29°20'48"N, 101°07'39"E−102°10'23"E, elevation 1,440–6,010 m) and Yunnan Province (24°13'16"N−26°30'19"N, 100°43'21"E−102°32'25"E, elevation 691–2,500 m) of China in 2021. Macroscopic characters, locations, altitudes, and dates were recorded, and photos of the fresh basidiomata were taken. Color codes indicated in the descriptions are from Kornerup and Wanscher (1978). The fresh materials were dried using an electric dryer at 45 °C for 8–10 h and stored thereafter as exsiccates in a sealable plastic bag. The dried specimens, along with the holotype of the newly described species, were deposited in the Herbarium of Hunan Normal University (MHHNU), Changsha, China.

Microscopic structures were observed with light microscopy using dried material revived in 5% KOH and stained with Congo Red when necessary. Basidiospores were mounted in Melzer’s reagent to test the amyloidity. The abbreviation (n/m/p) was used to describe basidiospores, where n was the number of basidiospores from m basidiomata of p collections. Dimensions for the basidiospores were provided using the notation (a)b–c(d). The range b−c contained a minimum of 90% of the measured values, and extreme values (a or d) were shown in parentheses. The letter Q was used for the length/width ratio of a basidiospore in side view, while Q_m_ indicated the average Q of all spores ± sample standard deviation (Bas 1969).

### ﻿DNA extraction, PCR amplification, and sequencing

Methods for genomic DNA extraction, PCR amplification, and sequencing followed those described in Su et al. (2022) and references therein. The following primer pairs were used for PCR amplification and sequencing: LR0R/LR5 was used to amplify the nrLUS region (Vilgalys and Hester 1990); ITS5/ITS4 for the ITS region (Gardes and Bruns 1993; White et al. 1990); EF1-983F/EF1-1567R for the *TEF1-α* region (Rehner and Buckley 2005); Am-6F/Am-7R for the RNA polymerase II (*RPB2*) region; and Am-β-tubulin F/Am-β-tubulin R for the second largest subunit of beta-tubulin (*TUB2*) region (Cai et al. 2014).

### ﻿Sequence alignment and phylogenetic analyses

The newly generated sequences in this study were deposited in GenBank, and additional sequences were retrieved from previously published articles and from GenBank (Table 1). Among these sequences, the majority belong to all the sect. Amanita species from China, while others are the relevant sequences of two new sequences retrieved through BLAST search from GenBank (Table 1). The phylogenetic trees of nrLUS and ITS were constructed for 52 and 55, respectively. The combined dataset (nrLUS, ITS, *TEF1-α*, *RPB2*, and *TUB2*) included 45 sequences, which at least four of these gene loci (Table 1). Sequences of all gene fragments were separately aligned with MUSCLE v3.8.31 (Edgar 2004) and manually edited using BioEdit v7.0.5 (Hall 1999). The concatenated matrix (including nrLUS, ITS, *TEF1-α*, *RPB2*, and *TUB2*) was constructed using SequenceMatrix 1.7.8 (Vaidya et al. 2011). The maximum likelihood (ML) and Bayesian inference (BI) were used on the concatenated alignment for phylogenetic tree inference. The ML analysis was performed using RAxML 7.2.6 (Stamatakis 2006) under the GTRGAMMAI model, executing rapid bootstrapping with 1000 replicates. Other parameters used default settings. The BI was performed in MrBayes v3.2 (Ronquist et al. 2012); four Markov Chain Monte Carlo (MCMC) chains were run simultaneously for one million generations under best partition schemes, and evolutionary models were selected using MrModeltest 2.3 (Nylander 2004). Runs were automatically terminated when the average standard deviation of split frequencies fell below 0.01. Subsequently, sampled trees were summarized, and posterior probabilities were obtained by discarding the first 25% of generations as burn-in. The phylograms from ML and BI analyses were visualized with FigTree v1.4.3 (Rambaut 2009) and then edited in Adobe Illustrator CS6.

**Table 1.** Taxa and GenBank accession numbers of sequences used in the phylogenetic analyses.

Taxon	Specimen No.	Location	nrLSU	ITS	*rpb2*	*tef1-α*	*tub2*	References
*A.albocreata*	RET547-7	USA	—	KU248128	—	—	—	Unpublished
*A.albocreata*	AMALB1	India	—	MH930873	—	—	—	Unpublished
*A.alpinicola*	CLC2376	USA	—	KR152655	—	—	—	Cripps et al. (2017)
*A.alpinicola*	RET888-8	USA	—	OL584339	—	—	—	Unpublished
*A.altipes*	HKAS91125	Sichuan, China	MH486367	MH508254	MH485862	MH508669	MH485401	Cui et al. (2018)
*A.altipes*	MHHNU32286	Yunnan, China	ON139026	ON131731	ON229622	ON229662	ON125308	Su et al. (2023)
*A.borealis*	BJTC-L169	Beijing, China	OR042384	OR058500	OR051503	OR051532	—	Zhou et al. (2023)
*A.breckonii*	MO402943	USA	—	OP470111	—	—	—	Unpublished
*A.breckonii*	NY00066695	USA	KJ535440	KJ535439	—	—	—	Unpublished
*A.collariata*	MHHNU31095	Hunan, China	OM955204	OM955206	OM949814	OM949813	OM949821	Su et al. (2022)
*A.concentrica*	FB-24901	Japan	—	AB080783	—	—	—	Oda et al. (2002)
*A.concentrica*	HKAS87061	Yunnan, China	KR824785	MH508327	KR824794	KR824827	MH485477	Cui et al. (2018)
*A.elata*	HKAS83449	Yunnan, China	MH486486	MH508334	MH485965	MH508763	MH485488	Cui et al. (2018)
*A.farinosa*	MHHNU32693	Hunan, China	ON139027	ON131732	ON229623	ON229663	ON125309	Su et al. (2023)
*A.farinosa*	RET534-10	USA	KU186823	—	—	—	—	Unpublished
***A.flavomelleiceps***	**MHHNU33119**	**Sichuan, China**	**PQ330908**	**PQ326880**	**PQ356792**	**PQ356798**	**PQ356795**	**This study**
*A.flavopantherina*	HKAS82613	Yunnan, China	MH486519	MH508355	MH485989	MH508795	MH485512	Cui et al. (2018)
*A.flavopantherina*	MHHNU32267	Yunnan, China	ON139030	ON131735	ON229626	ON229666	ON125312	Su et al. (2023)
*A.gemmata*	MHM116	Mexico	—	EU569282	—	—	—	Unpublished
*A.griseopantherina*	HKAS82340	Sichuan, China	MH486571	MH508383	MH486035	MH508840	MH485554	Cui et al. (2018)
*A.griseopantherina*	HKAS83560	Tibet, China	MH486573	MH508385	—	MH508842	MH485556	Cui et al. (2018)
*A.ibotengutake*	FB-30969	Japan	—	AB080988	—	—	—	Oda et al. (2002)
*A.ibotengutake*	HKAS83269	Jilin, China	MH486590	—	MH486051	MH508858	MH485570	Cui et al. (2018)
*A.melleiceps*	FB-30953	Japan	—	AB015688	—	—	—	Oda et al. (2002)
*A.melleiceps*	MHHNU32769	Hunan, China	ON139070	ON131770	ON262928	ON262914	ON125352	Su et al. (2023)
*A.melleialba*	HKAS83446	Yunnan, China	KR824767	MH508430	KR824792	KR824813	MH485603	Cui et al. (2018)
*A.melleialba*	MHHNU31481	Hunan, China	ON139031	ON131736	ON229627	ON229667	ON125313	Su et al. (2023)
*A.mira*	HKAS91953	Yunnan, China	MH486646	MH508437	—	—	—	Cui et al. (2018)
*A.muscaria*	MB-001171	Germany	MH486652	MH508442	MH486101	MH508909	MH485617	Cui et al. (2018)
*A.muscaria*	MHHNU32652	Inner mongolia, China	ON139034	ON131739	ON229630	ON229670	ON125316	Su et al. (2023)
*A.orientigemmata*	HKAS80978	Yunnan, China	MH486708	MH508469	MH486140	MH508948	MH485649	Cui et al. (2018)
*A.orientigemmata*	MHHNU32700	Hunan, China	ON139063	ON131764	ON229657	ON229661	ON125345	Su et al. (2023)
*A.pantherina*	RET293-6	South Africa	MK204473	MK204464	—	—	—	Unpublished
*A.parvipantherina*	MHHNU32350	Yunnan, China	ON139038	ON131743	ON229634	ON229674	ON125320	Su et al. (2023)
*A.parvipantherina*	HKAS83663	Yunnan, China	MH486752	MH508499	MH486176	MH508981	MH485680	Cui et al. (2018)
***A.parvisychnopyramis***	**MHHNU11290**	Yunnan, China	**PQ326881**	**PQ326875**	**PQ356790**	**PQ356796**	**PQ356793**	**This study**
***A.parvisychnopyramis***	**MHHNU32953**	Yunnan, China	**PQ330907**	**PQ326876**	**PQ356791**	**PQ356797**	**PQ356794**	**This study**
*A.* sp. ‘*praecox*’	BW-PH082906-9	USA	HQ539725	—	—	—	—	Unpublished
*A.pseudopantherina*	HKAS80007	Yunnan, China	MH486777	MH508514	MH486191	MH509004	MH485698	Cui et al. (2018)
*A.pseudopantherina*	MHHNU31028	Yunnan, China	ON139039	ON131744	ON229635	ON22967	ON125321	Su et al. (2023)
*A.pseudosychnopyramis*	HKAS87999	Yunnan, China	KR824778	MH508530	KR824790	KR824824	MH485713	Cui et al. (2018)
*A.pseudosychnopyramis*	MHHNU32762	Zhejiang, China	OM753110	MZ 313998	OM777286	OM777284	OM777285	Su et al. (2023)
*A.rubrovolvata*	FB-30980	Japan	—	AB096053	—	—	—	Unpublished
*A.rubrovolvata*	MHHNU32524	Hunan, China	ON139046	ON131751	ON229642	ON229682	ON125328	Su et al. (2023)
*A.rufoferruginea*	HKAS84974	Yunnan, China	MH486843	MH508580	MH486253	MH509069	—	Cui et al. (2018)
*A.rufoferruginea*	MHHNU31887	Hunan, China	ON139074	ON131774	ON262932	ON262918	ON125356	Su et al. (2023)
*A.siamensis*	HKAS83681	Yunnan, China	MH486866	MH508593	MH486273	MH509089	MH485774	Cui et al. (2018)
*A.siamensis*	MHHNU30976	Hunan, China	ON139076	ON131775	ON262934	ON262920	ON125358	Su et al. (2023)
*A.sinensis*	HKAS100492	Fujian, China	MH486867	MH508594	MH486274	MH509090	MH485775	Cui et al. (2018)
*A.sinensis*	MHHNU8585	Hunan, China	ON139047	MK239263	ON229643	ON229683	ON125329	Su et al. (2023)
*A.subfrostiana*	HKAS32513	Yunnan, China	AF024477	—	—	—	—	Weiss et al. (1998)
*A.subfrostiana*	HKAS58847	Yunnan, China	JN941161	JN943172	JQ031119	KR824806	MH485800	Cui et al. (2018)
*A.subglobosa*	HKAS12009	Sichuan, China	AF024478	—	—	—	—	Weiss et al. (1998)
*A.subglobosa*	MHHNU32538	Hunan, China	ON139051	ON131753	ON229647	ON229687	ON125333	Su et al. (2023)
*A.subparcivolvata*	MHHNU32849	Hunan, China	OM955209	OM955210	OM949817	—	OM949824	Su et al. (2023)
*A.subparcivolvata*	MHHNU32907	Hunan, China	OM955205	OM955216	OM949818	—	OM949825	Su et al. (2022)
*A.subparvipantherina*	HKAS56986	Yunnan, China	KR824776	—	—	—	—	Ariyawansa et al. (2015)
*A.subparvipantherina*	MHHNU32361	Yunnan, China	ON139057	ON131759	ON229653	ON229659	ON125339	Su et al. (2023)
*A.sychnopyramis*	HKAS83454	Yunnan, China	MH486927	—	—	—	—	Cui et al. (2018)
*A.sychnopyramis*	MHHNU32801	Zhejiang, China	ON139062	ON131763	ON229656	ON229693	ON125344	Su et al. (2023)
**Outgroups**								
*A.battarre*	HKAS101399	Sichuan, China	MH486381	MH508261	MH485874	MH508683	MH485411	Cui et al. (2018)
*A.incarnatifolia*	HKAS100593	Anhui, China	MH486594	MH508400	MH486056	MH508862	MH485575	Cui et al. (2018)
*A.liquii*	HKAS58885	Yunnan, China	MH486627	MH508426	MH486077	MH508885	MH485593	Cui et al. (2018)
*A.longistriata*	HKAS68331	Yunnan, China	MH486631	MH508428	MH486081	MH508888	MH485595	Cui et al. (2018)

New species were highlighted in bold text.

### ﻿Toxin detection and LC-MS/MS conditions

The extraction procedure, liquid chromatography tandem mass spectrometry (LC-MS/MS) instrument together with the separation column, gradient elution, and MRM condition were the same as described previously (Zhang et al. 2022). Toxin content in the mushroom extract was estimated using standards of isoxazole derivatives (IBO and MUS) (Alta Scientific Co., Ltd., Tianjin, China).

## ﻿Results

### ﻿Phylogenetic analyses

**Figure 1.**
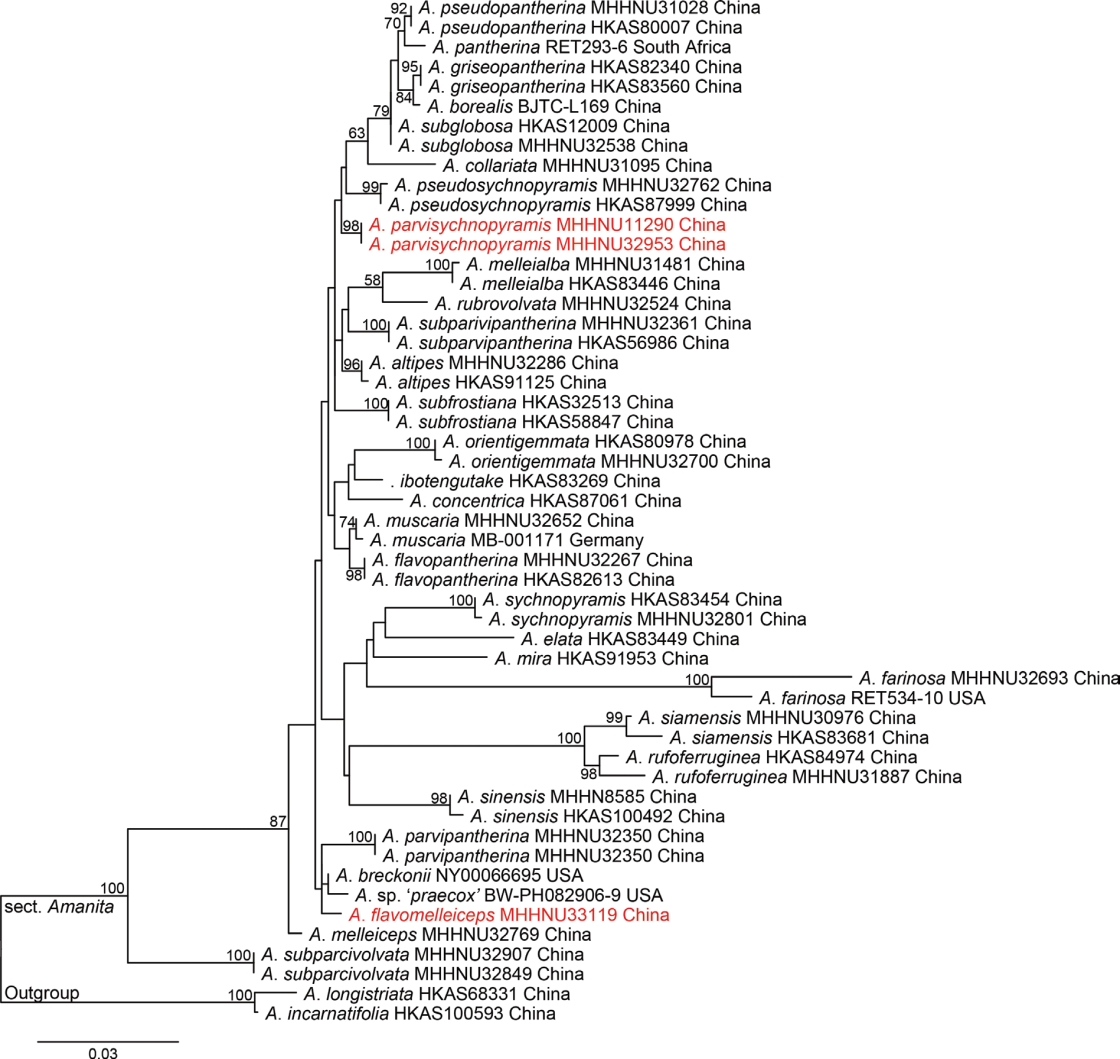
Phylogenetic tree inferred from maximum likelihood (ML) analysis based on the nrLSU regions. Bootstrap values over 50% are reported on the branches. Species names in red color indicate new species.

**Figure 2.**
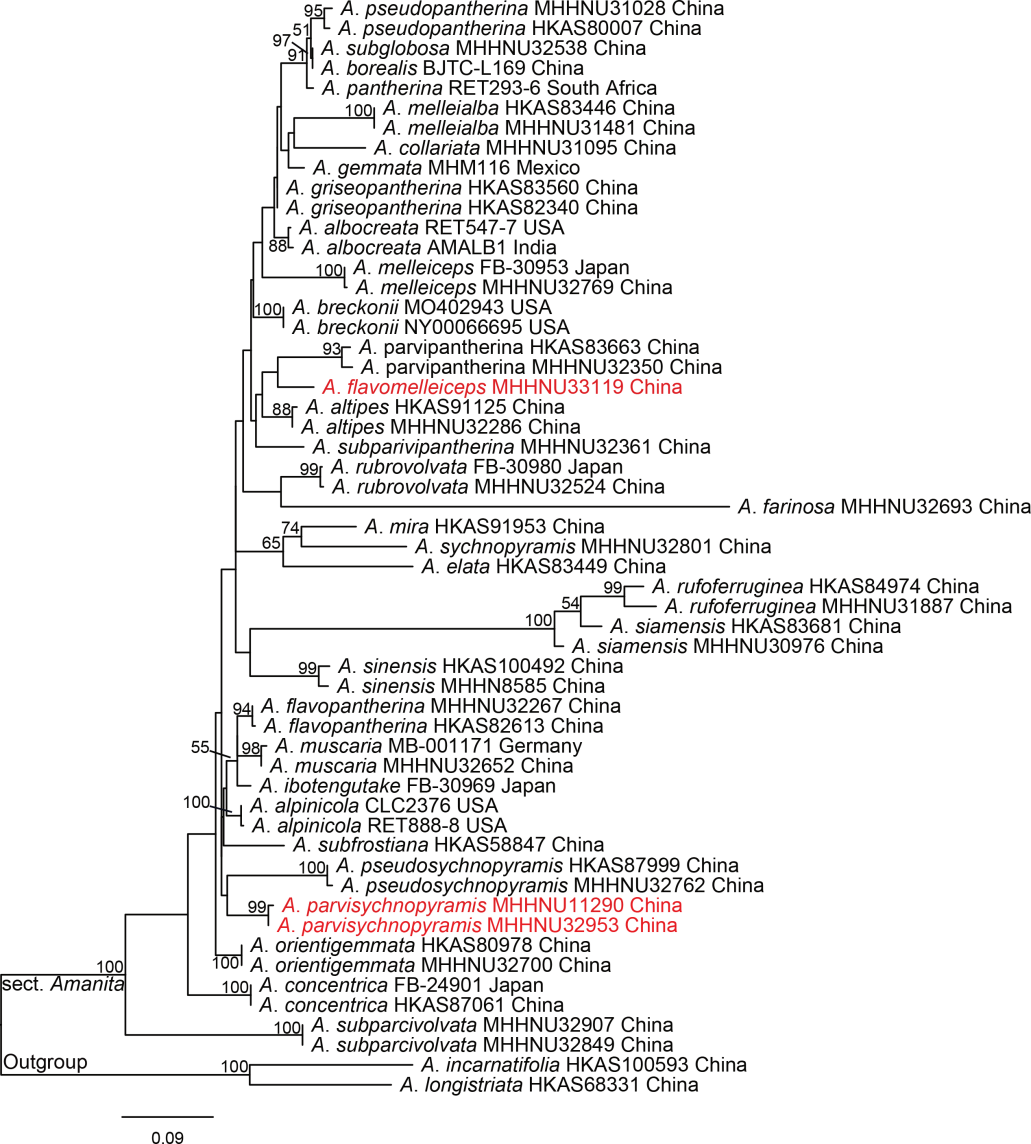
Phylogenetic tree inferred from maximum likelihood (ML) analysis based on the ITS regions. Bootstrap values over 50% are reported on the branches. Species names in red color indicate new species.

In the single gene loci dataset, the nrLSU and ITS data of *A.incarnatifolia* and *A.longistriata* as outgroups were analyzed together with the sequences of sect. Amanita, respectively. As a result of the alignment of nrLSU sequences ranging in length from 877 bp, 154 bp characters were parsimony-informative, and 194 bp variable characters were parsimony-uninformative. After excluding the ambiguous positions, the data matrix of the ITS region consisted of 842 bp, 445 bp were variable, and 319 bp were parsimony-informative characters. In ML analysis based on the nrLSU and ITS regions, they are shown in Figs 1, 2, respectively. Although the phylogenetic relationships among sect. Amanita in the nrLSU and ITS tree were not well resolved, the good support for distinct lineages in two new species (*A.flavomelleiceps* sp. nov. and *A.parvisychnopyramis* sp. nov.) allowed us to delineate species boundaries.

**Figure 3.**
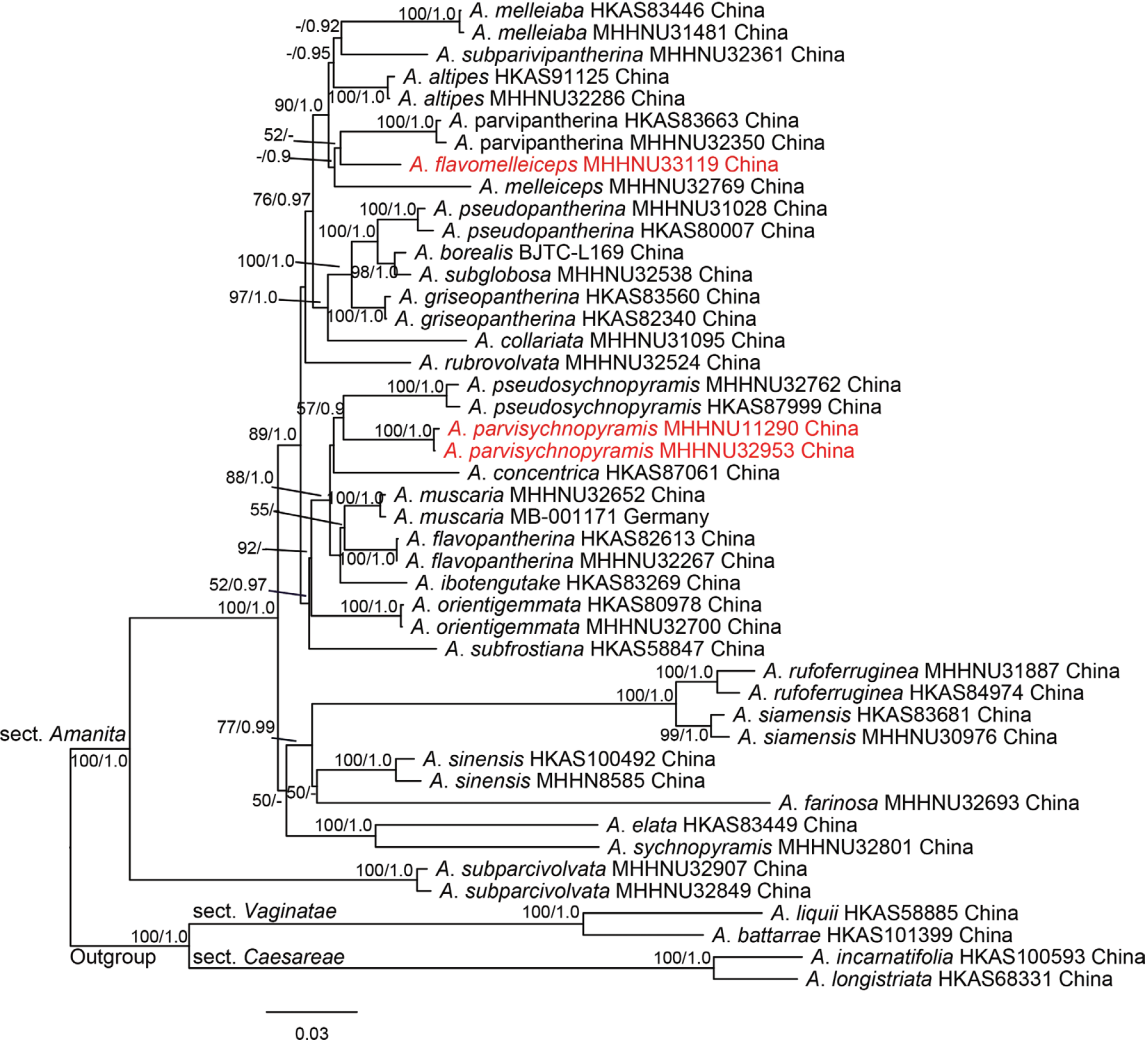
Phylogenetic tree inferred from maximum likelihood (ML) analysis based on the combined dataset (ITS, nrLSU, *RPB2*, *TEF1-α*, and *TUB2*). Bootstrap values over 50% and Bayesian posterior probabilities over 0.90 are reported on the branches. Species names in red color indicate new species.

In the combined dataset (nrLSU, ITS, *RPB2*, *TEF1-α*, and *TUB2*), the aligned lengths of the five gene loci were 938, 868, 652, 591, and 228 bp, respectively; contained 1009 parsimony-informative sites; and 1278 bp variable characters were parsimony-uninformative, and the introns of *RPB2*, *TEF1-α*, and *TUB2* were not removed. The topologies of ML and BI phylogenetic trees obtained in this study were nearly identical, and the tree obtained from the ML and BI analysis likelihood bootstraps and posterior probabilities support based on the dataset (Fig. 3). The target species, two new species, and representative samples from section Amanita were clustered into one distinctive clade with high support values, suggesting that the new species belong to A.sect.Amanita. Phylogenetically, *A.parvisychnopyramis* sp. nov. and *A.flavomelleiceps* sp. nov. were clearly separated from the other taxa (Fig. 3). They were therefore described here as new species, and sequences were deposited in GenBank.

### ﻿Taxonomy

#### ﻿ Amanita flavomelleiceps

Taxon classificationFungiAgaricalesAmanitaceae

Y.T. Su & Z.H. Chen sp. nov.

3193C246-2750-5491-85F0-8EFACABBD52D

855762

Figs 4 , 5

##### Etymology.

*flavomelleiceps*, from *flavo* = yellow, and *melleiceps* from *Amanitamelleiceps*, is proposed because this species is similar to *Amanitamelleiceps* but has yellowish, felted volval remnants on the pileus.

##### Holotype.

China • Sichuan Province, Ganzi Tibetan Autonomous Prefecture, Wuxuhai, altitude 2600 m 10 August 2021, Z.H.Chen, MHHNU 33119 (GenBank accession no. nrLSU: PQ330908; ITS: PQ326880; *rpb2*: PQ356792; *tef1-α*: PQ356798; *TUB2*: PQ356795).

##### Description.

**Figure 4.**
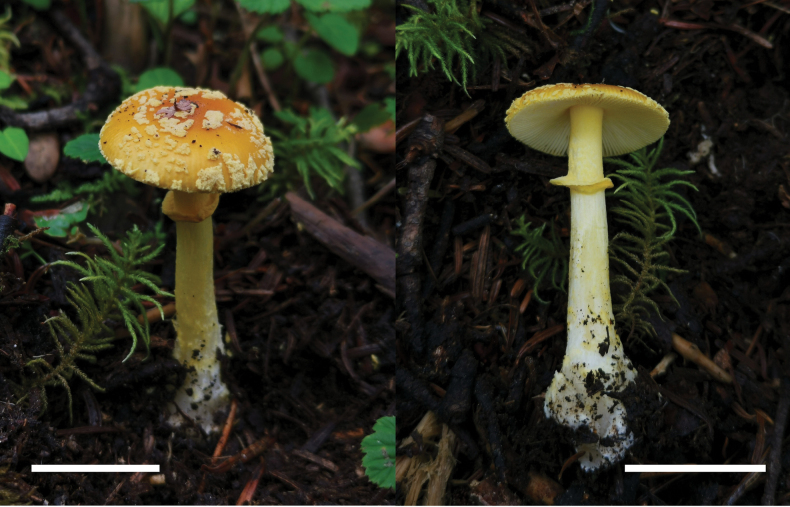
Fresh basidiomata of *Amanitaflavomelleiceps* (MHHNU 33119). Scale bar: 2 cm.

Basidioma (Fig. 4). small to medium. Pileus 2.5–5 cm diameter, convex to plano-convex, lacking a depression or umbo at center, yellowish (3A3–5), yellow (4A6–8), to yellowish brown (5C7–8), often darker at center and becoming paler towards margin; volval remnants on pileus verrucose to felted, 5–7 mm diameter, dirty yellow (3A2–4), randomly arranged; margin striate (0.37–0.41 R); trama white (1A1), unchanging. Lamellae free, cream (3A1), crowded; lamellulae truncate, plentiful. Stipe 5.5–9 cm long × 0.5–1 cm diameter, cylindric and slightly tapering upwards, with apex slightly expanded, dirty white (2B1) to white (1A1), sometimes with yellowish (3A2–3) tinge, covered with yellowish (3A3–5) fibrils; hollow in center; basal bulb subglobose, 0.7–1.5 cm diameter, white (1A1) to dirty white (2B1); volval remnants on stipe base floccose to granular and short limbate volva on limit between stipe and basal bulb, yellowish (3A3–5) to cream (3A1). Annulus persistent, subapical to median, yellowish (3A2–5) to yellow (4A5–8), membranous. Odor indistinct.

**Figure 5.**
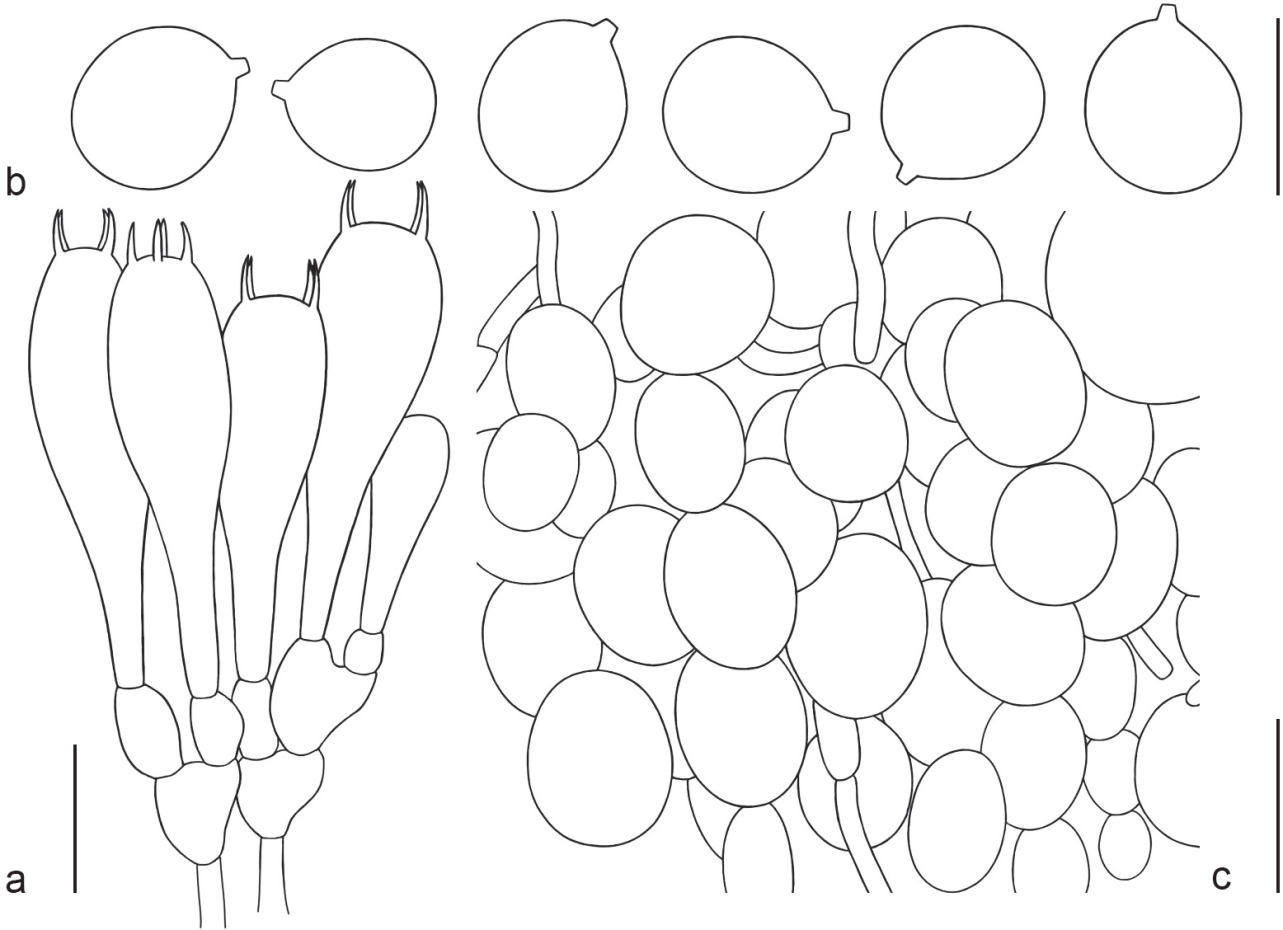
Microscopic features of *Amanitaflavomelleiceps* (MHHNU 33119) **a** hymenium and subhymenium **b** basidiospores **c** longitudinal section of volval remnants on pileus. Scale bar: 20 μm (**a**); 10 μm (**b**); 40 μm (**c**).

Microstructure (Fig. 5). Lamellar trama bilateral. Mediostratum 30–50 μm wide, composed of ellipsoid to clavate inflated cells (25–120 × 15–45 μm); filamentous hyphae fairly abundant, 3–7 μm wide; vascular hyphae scarce. Lateral stratum composed of ellipsoid to cylindrical inflated cells (20–70 × 15–30 μm), diverging at an angle of ca. 30°–40° to the mediostratum; filamentous hyphae abundant, 3–5 μm wide. Subhymenium (Fig. 5a) 40–60 μm thick, with 2–3 layers of subglobose to ellipsoid or irregular cells, 11–25 × 9–20 μm. Basidia (Fig. 5a) 40–60 × 14–16 μm, clavate, 4-spored; sterigmata 3–6 μm long; basal septa lacking clamps. Basidiospores (Fig. 5b) [110/4/1] (7.0–) 8.0–10 (–11.5) × (6.0–) 7.0–9.0 μm, Q = 1.0–1.22, Q_m_ = 1.06 ± 0.006, mostly subglobose, sometimes broadly ellipsoid, inamyloid, colorless, thin-walled, smooth; apiculus small. Lamellar edge appearing as a sterile strip up to 40–80 μm wide in side view, composed of broadly ellipsoid to long ellipsoid inflated cells (10–25 × 8–15 μm), single and terminal or in chains of 2–3, thin-walled, colorless; filamentous hyphae 2–4 μm wide, irregularly arranged or running parallel to lamellar edge. Pileipellis 110–180 μm thick; suprapellis up to 60–90 μm thick, gelatinized, composed of radially arranged, thin-walled, colorless or sometimes yellow-brown filamentous hyphae 2–6 μm wide; subpellis up to 50–70 μm thick, composed of radially and compactly arranged, filamentous hyphae 2–7 μm wide, yellowish or yellow-brown; vascular hyphae scarce. Volval remnants on pileus (Fig. 5c) composed of more or less vertically arranged elements; filamentous hyphae scarce, 2–7 μm wide, colorless, thin-walled, branching, anastomosing; inflated cells very abundant to dominant, subglobose or ellipsoid to broadly ellipsoid, 26–85 × 17–55 μm, colorless, thin-walled (≤ 0.05 μm), terminal or in chains of 2–3; vascular hyphae scarce. Interior of volval remnants on the stipe base composed of irregularly arranged elements; filamentous hyphae very abundant, 2–6 μm wide, colorless, thin-walled, branching, anastomosing; inflated cells scarce, subglobose to broadly ellipsoid (26–85 × 17–55 μm). Stipe trama composed of longitudinally arranged, clavate terminal cells, 28–130 × 12–80 μm; filamentous hyphae abundant, 2–5 μm wide; vascular hyphae scarce. Annulus dominantly composed of radially arranged elements; filamentous hyphae very abundant, 2–5 μm wide, colorless, thin-walled, branching, anastomosing; inflated cells scattered to fairly abundant, fusiform to elongate, 20–82 × 12–25 μm. Clamps absent in all parts of the basidioma.

##### Habitat.

Solitary to scattered on soil in broadleaved forests with Fagaceae; basidioma occurring in summer.

##### Distribution.

Currently known from southwestern China, but likely occurs more widely in the region with similar vegetation.

##### Commentary.

*Amanitaflavomelleiceps* sp. nov. can be easily confused with *A.altipes* Zhu L. Yang, M. Weiss & Oberw, *A.muscaria*, and *A.melleiceps* in their similar appearance and habitats. *Amanitaaltipes* was described from China by Yang et al. (2004). It has a bigger basidioma with floccose volval remnants and filamentous hyphae abundant to very abundant, and superior annulus (Cui et al. 2018). *Amanitamuscaria* is described from Europe (Gilbert 1940, 1941) and subsequently reported from China (Yang 2005, 2015). It is characterized by its relatively more robust basidioma with a pileus ca. 5–15 cm, white to dirty-white volval remnants on the pileus, and relatively bigger basidiospores (9.0–12.5 × 7.0–8.5 μm) (Cui et al. 2018). *Amanitamelleiceps* was described from Japan by Hongo (1966) and subsequently reported from China (Cui et al. 2018; Yang 2005, 2015). It differs in its honey-yellow pileus, patchy volval remnants on pileus, absent annulus, and relatively narrower basidia (40–55 × 8–11 μm) and basidiospores (8.5–10.5 × 6.0–7.5 μm) (Yang 2005, 2015; Cui et al. 2018).

#### ﻿ Amanita parvisychnopyramis

Taxon classificationFungiAgaricalesAmanitaceae

Y.T. Su & Z.H. Chen sp. nov.

35031905-BDD4-5EC5-A74E-B0A1638E8483

855761

Figs 6 , 7

##### Etymology.

*Parvisychnopyramis*, from *parvi* = small and *sychnopyramis* from *Amanitasychnopyramis*, is proposed because this species is similar to *A.sychnopyramis* but has smaller basidioma and basidiospores.

##### Holotype.

China • Yunnan Province, Chuxiong Yi Autonomous Prefecture, Zixi Mountain, in a mixed forest with Fagaceae, altitude 1900 m, 28 July 2021, Z.H. Chen and Y.T. Su, MHHNU 32953 (GenBank accession no. nrLSU: PQ330907; ITS = PQ326876; *RPB2*: PQ356791; *TEF1-α*: PQ356797; *TUB2*: PQ356794).

##### Description.

**Figure 6.**
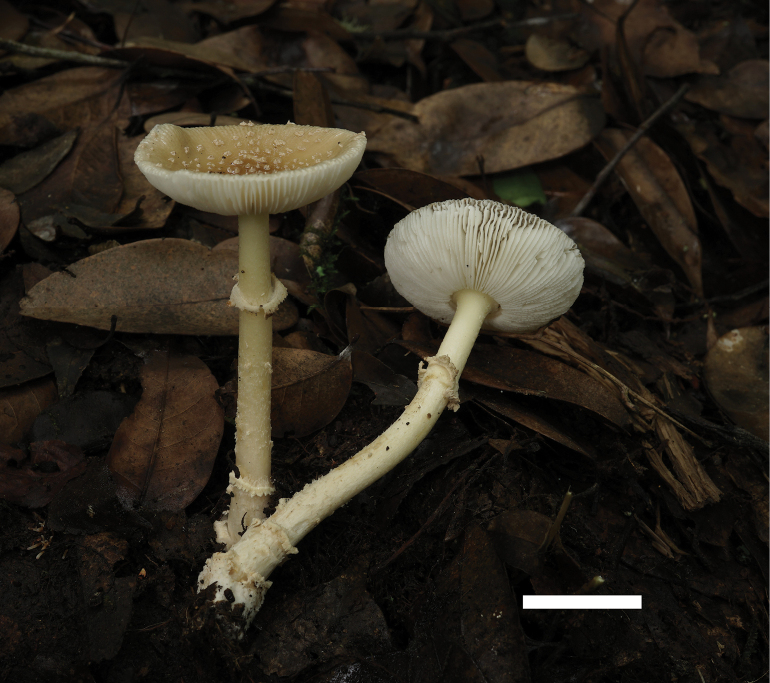
Fresh basidiomata of *Amanitaparvisychnopyramis* (MHHNU 32953). Scale bar: 2 cm.

Basidiomata (Fig. 6) Small-sized. Pileus 3–4 cm diameter, applanate, center often depressed, light brown (2B3–5) to brownish (3C3–4), with a brown center (4C5–8), becoming pale brownish (2B2–3) toward margin; margin striate (0.25–0.39 R); volval remnants conical to subconical, cream colored (3A1) to yellowish (1B3–4), radially and compactly arranged over the disk but easily removed; trama white (1A1), unchanging. Lamellae free, white (1A1); lamellulae truncate, evenly distributed. Stipe 7–8 cm long × 0.5–1 cm diam., dirty white (2B1) to white (1A1), subcylindrical and slightly tapering upwards, with apex slightly expanded, covered with cream (1A2) to yellowish (2A2) fibrils, often becoming floccose to patchy near basal bulb; context white (1A1), fistulose; basal bulb subglobose to ovate, 0.8–1.2 cm diam., volval remnants on stipe base collar-like, or shortly limbate, cream (3A1) to yellowish (1A3). *Annulus* present, superior to subapical, pale brownish (2B2–4) to dirty white (3A1), membranous, fragile. Odor indistinct.

**Figure 7.**
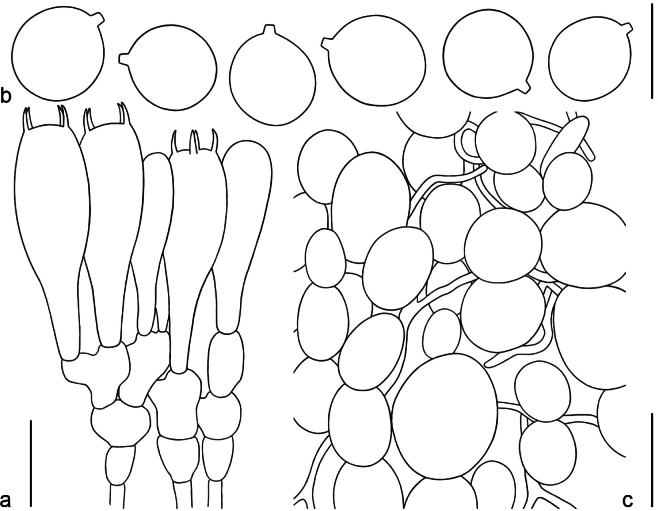
Microscopic features of *Amanitaparvisychnopyramis* (MHHNU 32953) **a** hymenium and subhymenium **b** basidiospores **c** longitudinal section of volval remnants on pileus. Scale bar: 20 μm (**a**); 10 μm (**b**); 40 μm (**c**).

Microstructure (Fig. 7), Lamellar trama bilateral. Mediostratum 30–40 μm wide, composed of abundant ellipsoid to long-ellipsoid inflated cells (20–95 × 10–40 μm); abundant filamentous hyphae, 2–4 μm wide; vascular hyphae scarce. Lateral stratum composed of abundant clavate to long-clavate inflated cells (27–65 × 11–25 μm), diverging at an angle of ca. 30°–45° toward mediostratum; filamentous hyphae abundant, 3–5 μm wide. Subhymenium (Fig. 7a) 25–40 μm thick, with 2–3 layers of subglobose to ellipsoid or irregular cells, 11–27 × 9–25 μm. Basidia (Fig. 7a) 35–56 × 11–15 μm, clavate, 4-spored; sterigmata 3–6 (–7) μm long; basal septa lacking clamps. Basidiospores (Fig. 7b) [100/3/2] (8.5–) 9.0–11.0 × 8.0–10.0 μm, Q = 1.0–1.16 (–1.22), Q_m_ = 1.10 ± 0.08, mostly subglobose and broadly ellipsoid, inamyloid, colorless, thin-walled, smooth; apiculus small. Lamellar edge appearing as a sterile strip, composed of subglobose, broadly ellipsoid to clavate inflated cells (12–25 × 7–15 μm), single and terminal or in chains of 2–3, thin-walled, colorless; filamentous hyphae abundant, 2–4 μm wide, irregularly arranged or running more or less parallel to lamellar edge. Pileipellis 100–125 μm thick; suprapellis up to 60–90 μm thick, gelatinized, composed of radially thin-walled, colorless, filamentous hyphae 2–6 μm wide; subpellis up to 50–75 μm thick, composed of radially and compactly arranged, filamentous hyphae 2–7 μm wide; vascular hyphae scarce. Volval remnants on pileus (Fig. 7c) composed of ± vertically arranged elements; filamentous hyphae scarce to fairly abundant, 2–6 μm wide, colorless, thin-walled, branching, anastomosing; inflated cells very abundant to dominant, subglobose, fusiform to ellipsoid (15–62 × 8–55 μm), colorless, thin-walled (≤ 0.05 μm), terminal or in chains of 2–3; vascular hyphae scarce. Interior of volval remnants on stipe base dominantly composed of longitudinally arranged elements; filamentous hyphae very abundant, 2–6 μm wide, colorless, thin-walled, branching, anastomosing; inflated cells fairly abundant, subglobose to broadly ellipsoid or clavate (10–81 × 9–40 μm). Stipe trama composed of longitudinally arranged, clavate terminal cells, 10–171 × 8–50 μm; filamentous hyphae scattered to abundant, 2–6 μm wide; vascular hyphae scarce. Annulus dominantly composed of subradially arranged elements; filamentous hyphae abundant, 2–5 μm wide, colorless, thin-walled, branching, anastomosing; inflated cells fairly abundant, clavate to long ellipsoid (26–118 × 23–59 μm), colorless, thin-walled; vascular hyphae rare. *Clamps* absent in all tissues.

##### Habitat.

Solitary to scattered on soil in broadleaved forests with Fagaceae; basidioma occurring in summer.

##### Distribution.

Currently known from southwestern China, but likely occurs more widely in other areas with similar vegetation.

##### Additional specimen examined.

China • Yunnan Province: Chuxiong Yi Autonomous Prefecture, Zixi Mountain, in a subalpine forest, altitude 1600 m, 25 August 2022, Ping Zhang, MHHNU 11290.

##### Commentary.

*Amanitaparvisychnopyramis* sp. nov. also shows some similarities with species in China, including *A.collariata* Y.T. Su, Zhu L. Yang & Z.H. Chen, *A.parvipantherina*, and *A.sychnopyramis*. *Amanitacollariata* was recently described from China (Su et al. 2022), and this species is distinguished from *A.parvisychnopyramis* sp. nov. by the granular volval remnants on pileus, volval remnants on the collar-like stipe base, relatively smaller basidia (35–53 × 10–15 μm), and narrower basidiospores (10.0–11.5 × 7.0–9.0 μm) (Su et al. 2022). *Amanitaparvipantherina* was described from China by Yang et al. (2004) and subsequently reported from India (Bhatt et al. 2017) and China again (Yang 2005, 2015; Cui et al. 2018). It is characterized by its small- to medium-sized basidioma, verrucose to pyramidal volval remnants on the pileus, and broadly ellipsoid to ellipsoid basidiospores (8.5–11.5 × 6.5–8.5 μm) (Yang et al. 2004). *Amanitasychnopyramis* was described from Japan by Hongo (1971) and subsequently reported from China (Yang 2005, 2015; Cui et al. 2018). The species is characterized by medium-sized basidioma with a median annulus, volval remnants on the pyramidal pileus, and relatively smaller basidia (30–42 × 8–12 μm) and basidiospores (6.5–8.5 × 6.0–8.0 μm) (Yang 2005, 2015; Cui et al. 2018).

### ﻿Key to the species of Amanitasect.Amanita from China

**Table:** 

1	Annulus absent	**2**
–	Annulus present	**6**
2	Pileal surface grayish to pale brownish, volval remnants on pileus pulverulent, gray to brown-gray	***A.farinosa***
–	Pileal surface yellowish, yellow to orange-red; volval remnants on pileus cream to yellowish	**3**
3	Pileal surface orange-red to red at center; volval remnants on pileus conical to verrucose, yellow to yellowish	***A.subparcivolvata***
–	Pileal surface yellowish to yellow-brown	**4**
4	Volval remnants on pileus felted to conical	**5**
–	Volval remnants on pileus floccose to pulverulent	***A.elata***
5	Volval remnants on pileus pyramidal to conical; basidiospores smaller, 6.0–8.0 × 6.0–7.5 μm	***A.mira***
–	Volval remnants on pileus patchy to felted; basidiospores larger, 9.0–11.0 × 6.0–8.0 μm	***A.melleiceps***
6	Pileal surface white, dirty white to pale grayish	**7**
–	Pileal surface and volval remnants non-white to dirty white	**8**
7	Volval remnants on stipe pyramidal to verrucose, white to dirty white	***A.concentrica***
–	Volval remnants on stipe pulverulent to floccose, gray to dark gray	***A.sinensis***
8	Basidiospores globose to subglobose, occasionally broadly ellipsoid, Q = 1.0–1.28	**9**
–	Basidiospores broadly ellipsoid, ellipsoid to elongate, Q = 1.15–1.9	**16**
9	Pileal surface red to orange	**10**
–	Pileal surface gray-brown, brown, yellow-brown to yellow	**11**
10	Volval remnants on pileus yellowish to yellow; basidiospores, 8.5–11.0 × 8.0–10.0 μm; clamps present	***A.subfrostiana***
–	Volval remnants on pileus red to orange; basidiospores, 8–10 × 7–9 μm; clamps absent	***A.rubrovolvata***
11	Pileal surface yellow-brown; volval remnants pulverulent, reddish brown to brown; stipe surface covered with reddish brown to brown, pulverulent squamules	***A.rufoferruginea***
–	Pileal surface yellowish, gray-brown, brown to dark brown; volval remnants patchy, verrucose to pyramidal; stipe glabrous or covered with yellowish-gray to dark-gray squamules	**12**
12	Pileal surface yellowish to yellow, yellowish brown; volval remnants verrucose, floccose to felted, yellowish, yellow to dirty yellow	**13**
–	Pileal surface brownish, gray-brown, brown to dark brown; volval remnants pyramidal to conical, dirty white to graish	**14**
13	Stipe covered with minute, volval remnants on stipe patchy to verrucose; annulus superior to subapical; basidiospores wider, 8–10 × 8–9 μm	***A.altipes***
–	Stipe covered with fibrils, volval remnants on stipe base floccose to granular; annulus subapical to median; basidiospores narrower, 8–10 × 7–9 μm	***A.flavomelleiceps***
14	Volval remnants on stipe base often collar-like or shortly limbate; annulus superior	**15**
–	Volval remnants on stipe base verrucose, conical to pulverulent	***A.sychnopyramis***
15	Basidiospores relatively smaller, 8.0–10.0 × 7–8.5 μm	***A.pseudosychnopyramis***
–	Basidiospores relatively larger, 9–11 × 8–10 μm	***A.parvisychnopyramis***
16	Clamps present	**17**
–	Clamps absent	**22**
17	Pileal surface yellow, red to orange; volval remnants on stipe base often as warts arranged irregularly or in concentrated incomplete belts	**18**
–	Pileal surface brownish, brown to dark brown; volval remnants on stipe base often collar-like or shortly limbate	**19**
18	Pileal surface red to orange; volval remnants on pileus pyramidal, verrucose to conical; basidiospores relatively larger, 9–12 × 7–8.5 μm	***A.muscaria***
–	Pileal surface yellowish to yellow; volval remnants on pileus felted to patchy, sometimes pyramidal; basidiospores relatively smaller, 9–10.0 × 6–8 μm	***A.orientigemmata***
19	Basidioma relatively smaller; pileal surface relatively lighter, brownish to brown	**20**
–	Basidioma relatively larger; pileal surface relatively darker, brown to dark brown	**21**
20	Basidiospores relatively smaller, 8–10 × 6–7.5 μm	***A.ibotengutake***
–	Basidiospores relatively larger, 8.5–12 × 7–9.5 μm	***A.subglobosa***
21	Volval remnants on pileus yellow; annulus subapical to media	***A.flavopantherina***
–	Volval remnants on pileus white to dirty white; annulus apical to subapical	***A.griseopantherina***
22	Pileal surface orange-brown with olivaceous tinge; volval remnants on pileus yellowish brown to orange-brown, often with olivaceous tinge	***A.siamensis***
–	Pileal surface yellow, grayish brown, brownish to brown; volval remnants on pileus white, cream to gray	**23**
23	Pileal surface honey yellow to yellow at center	***A.melleialba***
–	Pileal surface grayish brown to brown	**24**
24	Basidioma relatively smaller, pileus 3–5 cm diam	**25**
–	Basidioma relatively larger, pileus 5–8 cm diam	**26**
25	Volval remnants on stipe base collar-like, or shortly limbate, dirty white (3A1) to yellowish brown; annulus superior to subapical	***A.collariata***
–	Volval remnants on stipe base floccose to granular, white, cream to brownish; annulus subapical	***A.parvipantherina***
26	Basidiospores relatively broader, 9–12 × 7–9 μm	***A.pseudopantherina***
–	Basidiospores relatively narrower, 9–12 × 6.5–8 μm	***A.subparvipantherina***

### ﻿Analysis of toxins

**Figure 8.**
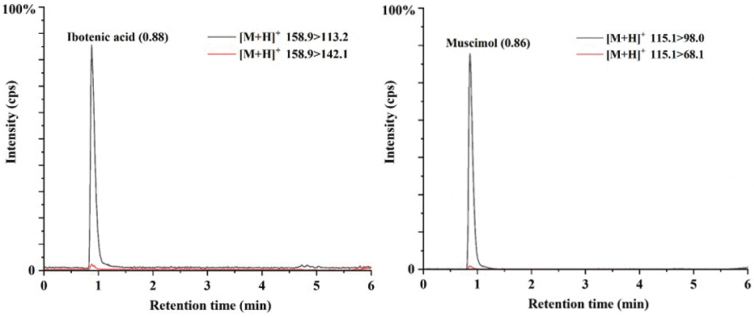
Multiple reaction monitoring (MRM) chromatograms of ibotenic acid and muscimol in *A.parvisychnopyramis*.

In this study, the protonated molecular ions of ibotenic acid ([M + H]^+^ = 158.9) and muscimol ([M + H]^+^ = 115.1) were chosen as the parent ions into two daughter ions that were used as the quantitative ion (ibotenic acid of 113.2, muscimol of 98.0) and qualitative ion (ibotenic acid of 142.1, muscimol of 68.1). The qualitative results show that *A.parvisychnopyramis* contained ibotenic acid and muscimol using LC-MS/MS (Fig. 8). Meanwhile, the content ranges of ibotenic acid and muscimol in *A.parvisychnopyramis* are 0.71–4.04 g/kg and 2.55–2.68 g/kg, respectively. No corresponding intensity was identified from *A.flavomelleiceps*.

## ﻿Discussion

### ﻿Species delimitation

Species of Amanitasect.Amanita is distributed worldwide; circa 150 species have been accepted, and 28 taxa have been reported from China (Cui et al. 2018; Su et al. 2022; Zhou et al. 2023). *Amanitaflavomelleiceps* sp. nov. is characterized by its yellowish to yellow pileus covered with yellowish, verrucose to felted volval remnants, and subglobose basal bulb with shortly limbate and mostly subglobose basidiospores (8.0–10.0 × 7.0–9.0 μm, Q = 1.0–1.22, Q_m_ = 1.06 ± 0.006). The other new species, *A.parvisychnopyramis* sp. nov., represents a new toxic *Amanita* from China that can be recognized by its small basidioma, brownish pileus covered with subconical volval remnants, an ovate basal bulb with a cream limbate, and subglobose to broadly ellipsoid basidiospores (9–11.0 × 8.0–10.0 μm).

All phylogenetic analyses revealed that *A.flavomelleiceps* sp. nov. and *A.parvisychnopyramis* sp. nov. are supported as an independent lineage in A.sect.Amanita. In two single-gene loci ML trees, new species are weakly supported as distantly related to other species. In multi-locus phylogeny of sect. Amanita, *A.flavomelleiceps* sp. nov. is distantly related to *A.altipes*, *A.melleiceps*, and *A.melleialba* Zhu L. Yang, Q. Cai & Y.Y. Cui, *A.parvipantherina* and *A.subparvipantherina* Zhu L. Yang, Q. Cai & Y.Y. Cui (ML BS = 90%, BI PP = 1.0). However, *A.melleialba* (7.5–9.5 × 6.0–7.0 μm, Q = 1.29–1.58), *A.parvipantherina* (8.5–11.5 × 7.0–8.5 μm, Q = 1.13–1.47), and *A.subparvipantherina* (9.0–11.5 × 6.5–8.0 μm, Q = 1.28–1.5) are distinguished from *A.flavomelleiceps* sp. nov. by the ellipsoid to broadly ellipsoid basidiospores (Cui et al. 2018). *Amanitaparvisychnopyramis* sp. nov. is genetically distant from *A.pseudosychnopyramis*, and the two taxa form a clade (ML BS = 57%, BI PP = 0.9). However, *A.pseudosychnopyramis* presents some distinct morphological characteristics that clearly separate it from *A.parvisychnopyramis* sp. nov. *Amanitapseudosychnopyramis* was described from China by Ariyawansa et al. (2015) and is distinguished by its medium-sized basidiomata, conical to pyramidal grey to brownish-grey volval remnants on the pileus, and subglobose to broadly ellipsoid basidiospores (8.5–10 × 7.5–8.5 μm).

### ﻿Toxicity

For centuries, wild mushrooms have been consumed in large quantities and are popular in the human diet because of their matchless flavor and nutritional value, but eating wild mushrooms comes with the risk of poisoning (Chen et al. 2014). During picking, edible and poisonous mushrooms can easily be confused because of their morphological similarities. When in-depth investigations of neuropsychiatric mushroom poisoning incidents are made, researchers find that many mushroom poisoning incidents have been caused by members of A.sect.Amanita (Chen et al. 2016; Cui et al. 2018; Su et al. 2023). The toxins documented in those species are primarily ibotenic acid and muscimol, which can cause glutaminergic neurotoxicity (Diaz 2005; Chen et al. 2014, 2016). Until now, *A.cothurnata* G.F. Atk., *A.ibotengutake* T. Oda, C. Tanaka & Tsuda, *A.muscaria*, *A.pantherina*, and *A.strobiliformis* (Paulet ex Vittad.) Bertill have been known to contain those toxins and are distributed across North America and Europe (Chilton and Ott 1976; Spoerke and Rumack 1994; Oda et al. 2002; Gonmori et al. 2012; Poliwoda et al. 2014). In 2023, ten sect. Amanita species from China were reported to contain ibotenic acid and muscimol ranging from 0.6125 to 32.0932 and 0.0056 to 5.8685 g/kg dry weight, respectively (Chen et al. 2022; Su et al. 2023).

*Amanitaparvisychnopyramis* sp. nov. represents another new toxic species in China. This new species contains the most notorious neuropsychiatric toxins, ibotenic acid and muscimol, which range from 0.71 to 4.04 g/kg and 2.55 to 2.68 g/kg, respectively. *Amanitaflavomelleiceps* does not contain ibotenic acid and muscimol; however, this does not mean that it is safe for consumption. Su et al. (2023) investigated the presence of ibotenic acid and muscimol in most species of A.sect.Amanita in China. These two toxins were not detected in species causing neuropsychiatric poisoning events, such as *A.melleiceps* and *A.melleialba* Zhu L. Yang, Q. Cai & Y.Y. Cui, *A.orientigemmata* Zhu L. Yang & Yoshim. Doi, and *A.rufoferruginea* (Li et al. 2020, 2021, 2022). We speculate that they may contain unknown isoxazole derivative toxins, and we conclude that this area needs further study (Su et al. 2023). Therefore, it is important to avoid the consumption of all *Amanita* species to effectively prevent future poisoning incidents.

## Supplementary Material

XML Treatment for Amanita flavomelleiceps

XML Treatment for Amanita parvisychnopyramis

## ﻿Additional information

### Author ORCIDs

Fei Xu https://orcid.org/0000-0001-9219-1235

Peng-Tao Deng https://orcid.org/0000-0002-8755-7965

Meng-Meng Lai https://orcid.org/0009-0004-6519-6654

### Data availability

DNA sequence data that support the findings of this study have been deposited in the GenBank repository. Other data generated or analyzed during this study are included in this article.
